# In Vitro and In Vivo Evaluation of a Three-Dimensional Porous Multi-Walled Carbon Nanotube Scaffold for Bone Regeneration

**DOI:** 10.3390/nano7020046

**Published:** 2017-02-17

**Authors:** Manabu Tanaka, Yoshinori Sato, Mei Zhang, Hisao Haniu, Masanori Okamoto, Kaoru Aoki, Takashi Takizawa, Kazushige Yoshida, Atsushi Sobajima, Takayuki Kamanaka, Hiroyuki Kato, Naoto Saito

**Affiliations:** 1 Department of Orthopaedic Surgery, School of Medicine, Shinshu University, Asahi 3-1-1, Matsumoto, Nagano 390-8621, Japan; hhaniu@shinshu-u.ac.jp (H.H.); ryouyuma@shinshu-u.ac.jp (M.O.); kin29men@shinshu-u.ac.jp (K.A.); takashitak@shinshu-u.ac.jp (T.T.); gooddays83@yahoo.co.jp (K.Y.); soba@shinshu-u.ac.jp (A.S.); kam17@hotmail.co.jp(T.K.); hirokato@shinshu-u.ac.jp (H.K.); saitoko@shinshu-u.ac.jp (N.S.); 2 Graduate School of Environmental Studies, Tohoku University, Aoba 6-6-20, Aramaki, Aoba-ku, Sendai 980-8579, Japan; yoshinori.sato.b5@tohoku.ac.jp; 3 Institute for Biomedical Sciences, Shinshu University, Asahi 3-1-1, Matsumoto 390-8621, Japan; 4 High-Performance Materials Institute, Florida State University, 2005 Levy Avenue, Tallahassee, FL 32310, USA; mzhang3@fsu.edu

**Keywords:** tissue engineering, bone regeneration, carbon nanotube

## Abstract

Carbon nanotubes (CNTs) have attracted a great deal of attention for the biological and medical science fields because of their characteristic physical and biological properties. In this study, we investigated the capacity of the 3D porous CNT scaffold (CNT porous block; CNTp) for bone regenerative medicine. Surface observations using a scanning electron microscope (SEM), crystal depositions on the surface of CNTps immersed in simulated body fluid (SBF), and evaluations of protein adsorption and controlled releasing were conducted to assess physical properties. The cell proliferation and cell morphology were observed using SEM and fluorescent microscopy. CNTps were implanted into critical-size mouse calvarial defects and evaluated for their osteoconductive ability and in vivo controlled release of recombinant human BMP-2 (rhBMP-2). Interconnected porous HA ceramics (IP-CHAs) were used for comparison. CNTps have multiporous structures with interporous connections with networks of multiwalled CNTs. Crystals containing calcium and phosphate were deposited in CNTps and on the surface of the CNT networks by immersing CNTps in SBF. CNTps adsorbed more significantly and released protein more gradually than IP-CHAs. Preosteoblasts seeded onto CNTps filled pores with stretched actin filaments and filopodia. Compared with IP-CHAs, CNTps showed significantly higher cell proliferation, better osteoconduction, and more bone generation with rhBMP-2. In this study, CNTps demonstrated good osteoconductive ability, cell attachment and proliferation capacity, and growth factor retaining ability. CNTps have the potential not only as artificial bones for the treatment of bone defects, but also as scaffolds for regenerative medicine using tissue engineering approaches.

## 1. Introduction

Nonunions and large skeletal defects created by tumors, traumas, or congenital malformations are sometimes impossible to repair. In order to fill the aforementioned defects and promote bone regeneration, various approaches have been researched in clinical trials. Autologous bone grafts (autografts) are most commonly used, because they provide essential characteristics for bone regeneration, such as cells, growth factors, and scaffolds [1]. However, the disadvantages of autologous bone graft include limited availability of cancellous bone, residual pain, nerve injury, and cosmetic defects. Although allografts solve the above problems, their use may create an immune response or transmit viral diseases [2]. In order to address these issues, bone regenerative scaffolds made of synthetic biomaterials are now being used as bone graft substitutes [3], which are also called “artificial bones”. Type I collagen, demineralized bone matrix (DBM), poly lactic acid (PLA), poly glycolic acid (PGA), hydroxyapatite (HA), and tricalcium phosphate (TCP) have been used for materials and scaffolds [1]. Because bone graft is the second most common transplantation tissue [2], there is great demand for the development of scaffolds that can reduce the risk of autograft and allograft. One of the essential properties of bone regenerative scaffolds is their biocompatibility, which is described as an ability of a material to perform with an appropriate host response in a specific situation [4]. In addition, optimal bone regeneration scaffolds should be osteoconductive, which means osteogenic cells can attach, proliferate, and structure extracellular matrix on their surface [3]. In bone regenerative medicine, scaffolds made of HAs that hold approximately 50% of bone weight in various structures are used widely because of their osteocompatibility, and such materials are developed and reported to function well. Those with multipore structures measuring more than 100 μm in diameter are becoming mainstream [3]. Interconnected porous hydroxyapatite ceramics (IP-CHAs) are widely used as bone regenerative scaffolds because of their good biocompatibility, osteoconduction, primary stiffness, and pore size.

On the other hand, tissue engineering techniques using a combination of cells, growth factors, and scaffolds to regenerate various tissues have been researched broadly for their application in the regeneration of bones and cartilages [5]. The use of a drug delivery system [6] that retains and releases biomolecules gradually has been anticipated for scaffolds in tissue engineering. Biomolecules retained by scaffolds are proteins called growth factors, which include transforming growth factor (TGF-β), bone morphogenetic protein (BMP), insulin-like growth factor (IGF), fibroblast growth factor (FGF), and vascular endothelial growth factor (VGEF). These growth factors regulate bone formation by inducing osteoprogenitor cells to differentiate to osteogenic cells [7]. BMPs are known to induce the initial proliferation and differentiation of osteoprogenitor cells [8] and facilitate repair of critical-sized bone defects in animal models [9]. However, because BMPs rapidly degrade in the body [10], their in vivo application have been considered insufficient for clinical use. Thus, a long-term controlled release system of BMPs is deemed necessary. To develop an effective method, in vitro drug release assays [11] and in vivo evaluations of bone repair in mouse critical-sized bone defects [6] are broadly researched.

Carbon nanotubes (CNTs) are becoming attractive in the field of regenerative medicine because of their mechanical and biological properties [12] and processability [13]. CNTs are also known to have good biocompatibility and have been reported to accelerate cell differentiation. Moreover, reports have indicated that CNTs promote osteoblastic function but also inhibit osteoclasts [14,15]. CNTs are considered to be promising biomaterials for bone defect repair and bone-tissue engineering applications because of their unique behavior in the body. The most important concern for the use of CNTs as a biomaterial is safety. However, only few reports suggest that CNTs are toxic when applied in higher concentrations [16]. Good osteoregeneration has been reported by using CNTs as nanocomposites with polymers, collagen, PLA, and HA in bone regenerative medicine [17]. However, no research has been reported on the porous bone regenerative scaffold that is made of CNT alone. We compared the biocompatibility, osteoconduction, capacity to retain osteogenic proteins, and in vivo controlled-release of BMPs of 3D-shaped porous scaffold made of multi-walled carbon nanotube (MWCNT) to those of the IP-CHA for use in the field of bone regenerative medicine.

## 2. Results

### 2.1. Surface Observation by a Scanning Electron Microscopy

**Figure 1:**
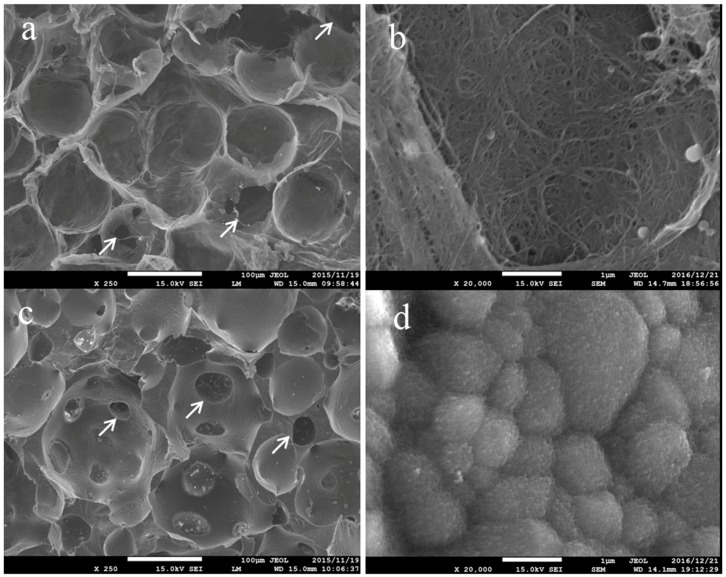
Scanning electron microscope (SEM) image of (**a**,**b**) carbon nanotube (CNT) porous block (CNTp) and (**c**,**d**) interconnected porous HA ceramic (IP-CHA). Original magnification is 250× for (**a**,**c**) and 20,000× for (**b**,**d**). White arrows: interporous connections.

CNTps have substantially uniform micro-pores of approximately 100 μm in diameter, which were interconnected randomly by small 30–50 μm pores (Figure 1a). The pore surface of CNTps were smooth and fibrous. MWCNTs were observed in higher (20,000×) magnification (Figure 1b). The roughness of the pore surface is in nanometer scale. IP-CHAs have pores of 50–250 μm in diameter with interpore connections (Figure 1c) and smooth pore surfaces with aligned granular HA crystals (Figure 1d).

### 2.2. Mechanical Strength

**Figure 2:**
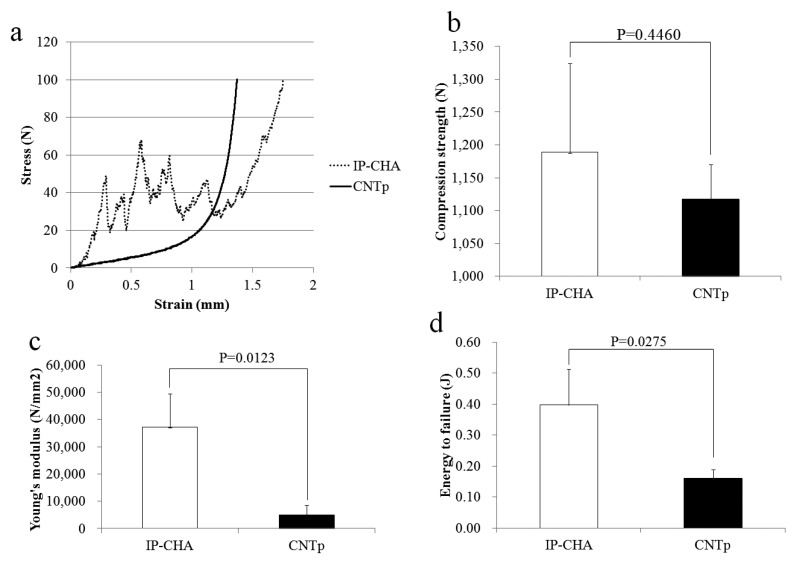
The stress-strain curves (**a**); maximum compressive strength (**b**); Young’s modulus (**c**); and energy to failure (**d**) of the IP-CHA and CNTp. Mean values were compared using Student’s *t* test (*n* = 3).

Figure 2 shows the stress-strain curves (a); maximum compressive strength (b); Young’s modulus (c); and energy to failure (d) of the IP-CHA and CNTp. The CNTp broke more smoothly than the IP-CHA. The maximum compressive strengths of the CNTp and IP-CHA showed no significant difference between the two groups (1117 ± 52.21 vs. 1188 ± 135.79 N, *p* = 0.4460). The IP-CHA showed a significantly higher Young’s modulus than the CNTp (37,144 ± 12,359 vs. 4973 ± 3576 N/mm^2^, *p* = 0.0123). The IP-CHA also showed a significantly higher energy to failure than the CNTp (0.397 ± 0.117 vs. 0.161 ± 0.028 J, *p* = 0.0275).

### 2.3. Crystal Deposition Analysis

**Figure 3:**
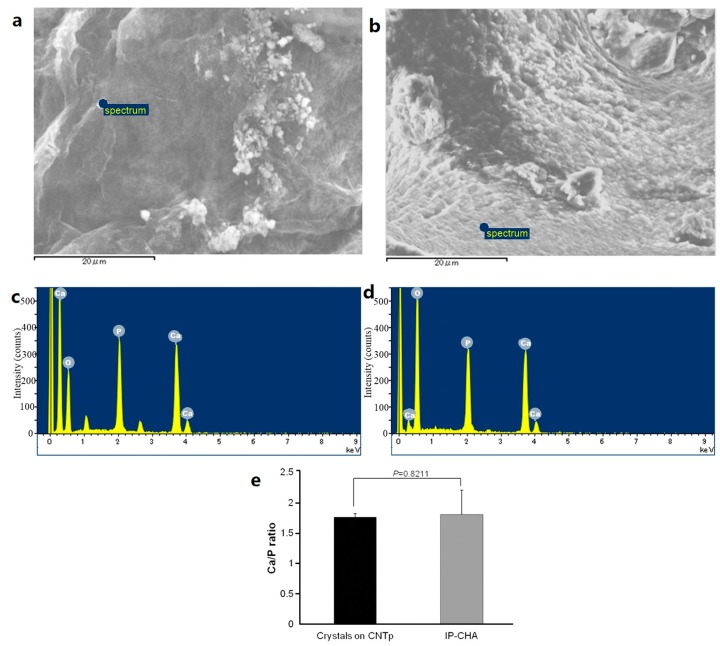
(**a**) SEM image of hydroxyapatite-like crystal formation on CNTp scaffolds; (**b**) SEM image of a non-treated IP-CHA; (**c**,**d**) EDS on marked area revealed Ca, P, O in the crystal on (**c**) CNTp and (**d**) the surface of non-treated IP-CHA; (**e**) Ca/P ratio of the crystals appeared on the surface of CNTp and IP-CHA. Mean values were compared using Student’s *t* test (*n* = 5).

After 14 days of immersing in revised SBF (r-SBF) [12], the micro-pore surfaces in CNTp were coated with aggregations of deposited nano-sized crystals (Figure 3a). In the analysis by energy-dispersive spectrometer (EDS, Inca X-Max 50, OXFORD INSTRUMENTS, Abingdon, Oxfordshire, UK), these aggregations contained calcium, phosphorus, and oxygen (Figure 3c). The ratio of elements was similar to that of non-treated IP-CHA that represents pure HA material. There were no significant differences in the Ca/P ratio between these aggregations in CNTp and non-treated IP-CHA (Figure 3e, 1.760 vs. 1.803, *p* = 0.8211).

### 2.4. Qualitative Protein Adsorption Profiles

**Figure 4:**
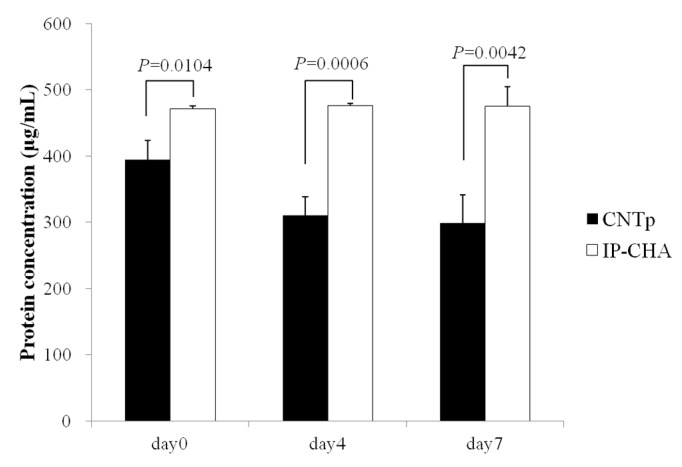
Qualitative protein adsorption profiles. Mean values were compared using Student’s *t* test (*n* = 3).

Each sample was immersed into 500 μL of 500 μg/mL Bovine Serum Albumin (BSA) solution (each solution contained 250 μg of BSA). On day 0 (one hour of immersion), CNTps showed significantly faster protein adsorption than IP-CHAs (*p* = 0.0104). On day 4, CNTps decreased more protein concentration in the solution compared to IP-CHAs (*p* = 0.0006). Similar data was obtained on day 7 (*p* = 0.0042). IP-CHAs did not show further decrease of protein concentration on days 4 and 7 compared to day 0 (Figure 4). Total amounts of protein that adsorbed to CNTps and IP-CHAs on day 7 were 100 μg and 12 μg, respectively. Considering the porosity of CNTps (99%) and IP-CHAs (75%), the CNTps absorbed 6.3 times more protein than IP-CHAs.

### 2.5. In Vitro Protein Releasing Assay

**Figure 5:**
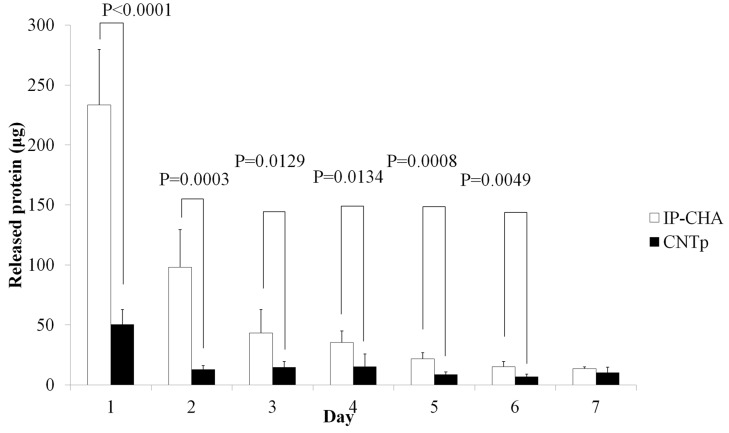
Qualitative protein released from scaffolds. Graph shows release of bovine serum albumin (BSA) from CNTp and IP-CHA. Mean values were compared using Student’s *t* test (*n* = 5).

The protein release was observed in both CNTp and IP-CHA groups. On day 1, CNTps released 10.1% of the total added protein, which is less than the IP-CHAs released (46.7%). In following days, CNTps and IP-CHAs released protein gradually but CNTps did not release 100% of total loaded protein as shown in Figure 5. Finally, they released 23.9% of total loaded protein after seven days compared to IP-CHA (92.2%).

### 2.6. Degradation Assay

**Figure 6:**
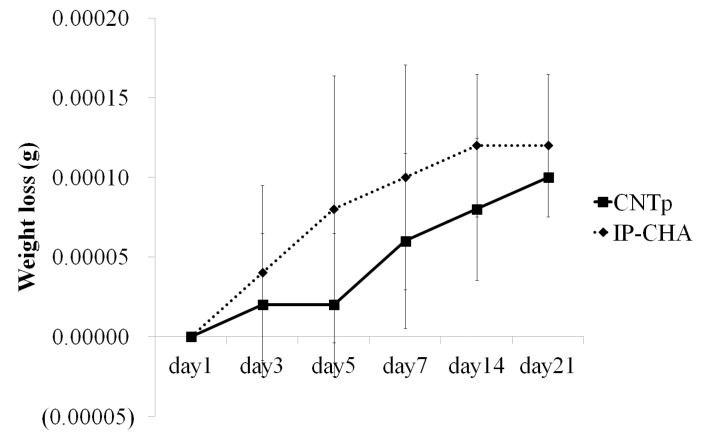
Degradation assay. Graph shows weight loss of CNTps and IP-CHAs immersed in PBS for 21 days. Mean values were compared using two-way ANOVA (*n* = 5).

Figure 6 demonstrates the percentage of mass lost over time. Both CNTps and IP-CHAs showed significant weight loss in 21 days (*p* = 0.0005 and *p* = 0.0002, respectively). However, each loss was very slight (within 0.0002 g/scaffold). IP-CHAs showed a slightly faster degradation rate than CNTps for 21 days (*p* = 0.0040).

### 2.7. Cell Morphology

**Figure 7:**
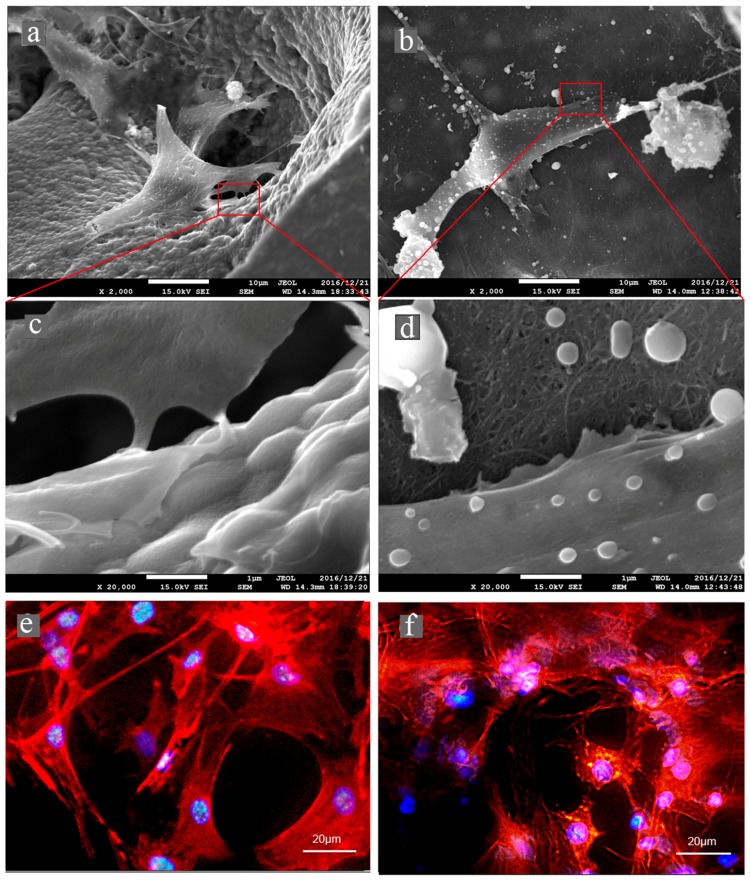
MC3T3-E1 cells spreading on IP-CHA and CNTp scaffolds. Cells on (**a**,**c**,**e**) IP-CHA or (**b**,**d**,**f**) CNTp. SEM images of (**a**,**c**) IP-CHA and (**b**,**d**) CNTp. Original magnification is 2000× for (**a**,**b**) and 20,000× for (**c**,**d**). Cells labeled for actin filaments (red) and nucleus (blue) of (**e**) IP-CHA and (**f**) CNTp.

Figure 7a–d shows the morphologies of MC3T3-E1 cells cultivated on the CNTps and IP-CHAs observed by FE-SEM. Cells were connected via cytoplasmic processes and expanded on the surface of both CNTps and IP-CHAs. Cells on CNTps expand more cytoplasmic processes (arrow). Figure 7e,f shows cell morphologies observed by a fluorescent microscope. Cells containing well-developed actin filaments and round cells attached to the inner surface of pores.

### 2.8. Cytotoxicity Determination

**Figure 8:**
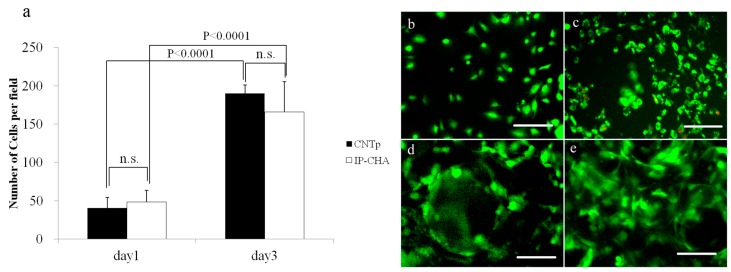
Cytotoxicity. Graph shows the number of cells per field counted on the surface of the CNTp and the IP-CHA (**a**). Mean values were compared using Student’s *t* test (*n* = 7). N.S.: Not Significant. Viable cells were stained with calcein AM (acetoxymethyl) and fluoresced green, while dead cells were stained with EthD-III and fluoresced red. Most of MC3T3-E1 cells were alive on CNTp (**b**,**c**) and IP-CHA (**d**,**e**) scaffolds at day 1 (**b**,**d**) and day 3 (**c**,**f**). White bars: 100 µm.

On day 1, the mean number of cells in a 20× field of CNTp and IP-CHA surface were 40.6 and 48.6, respectively, with no significant difference. On day 3, these were 190.3 and 165.7, respectively, with no significant difference. However, each scaffold showed significantly higher cell proliferation on day 3 than on day 1 (*p* < 0.0001 and *p* < 0.0001, respectively) as shown in Figure 8a. In the live/dead cell staining, visual inspection was consistent with unimpeded viability (Figure 8b–e).

### 2.9. Alkaline Phosphatase Activity Assay

**Figure 9:**
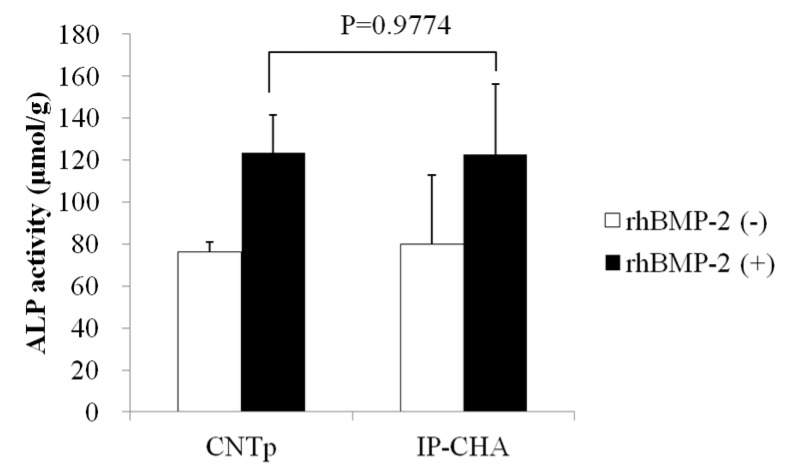
Alkaline Phosphatase (ALP) activity. Graph shows the ALP activity of the MC3T3-E1 cells cultured in the presence or absence of rhMP-2 on the surface of the CNTp and the IP-CHA. Mean values were compared using Student’s *t* test (*n* = 4).

After seven days, cells on CNTps and IP-CHAs showed significantly increased Alkaline Phosphatase (ALP) activities. There was no significant difference between the two groups (*p* = 0.9774) (Figure 9).

### 2.10. Bone Regeneration of Mouse Critical-Sized Calvarial Defects

**Figure 10:**
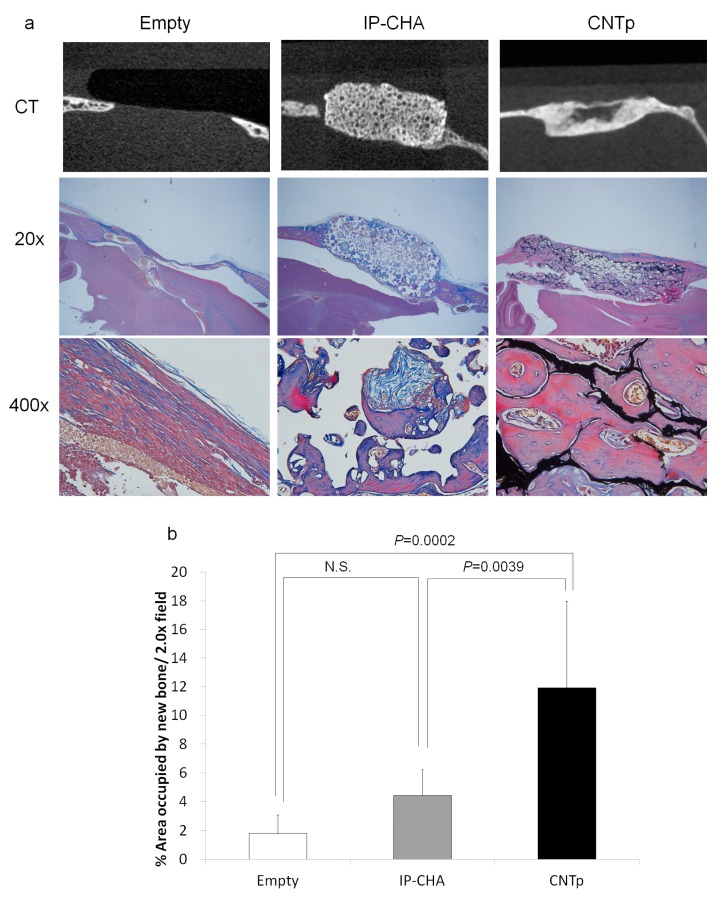
(**a**) Micro-CT and histological image of empty control, IP-CHA, and CNTp scaffold implanted in the calvarial defects of ddY mice for 12 weeks; (**b**) A bar diagram showing the newly formed bone area/20× field in the calvarial defect of ddY mice implanted with each scaffold for 12 weeks. Mean values were compared using one-way ANOVA followed by Tukey’s post hoc test (*n* = 7). N.S.: Not Significant.

Little bone regeneration occurred in the empty group. On the other hand, regeneration occurred in blue-stained collagen fibers, red- or purple-stained woven bones, and mature bones, filling in the pores of CNTp and IP-CHA scaffolds. Vascularizations were also observed in the newly formed bones (Figure 10a). By image analysis, the CNTp group contained more newly formed bone in a 20× field than the empty and IP-CHA group (*p* = 0.0002 and *p* = 0.0039, respectively.) (Figure 10b).

### 2.11. Bone Regeneration of Mouse Critical-Sized Calvarial Defects in Combination with Recombinant Human Bone Morphogenetic Protein-2 (rhBMP-2)

**Figure 11:**
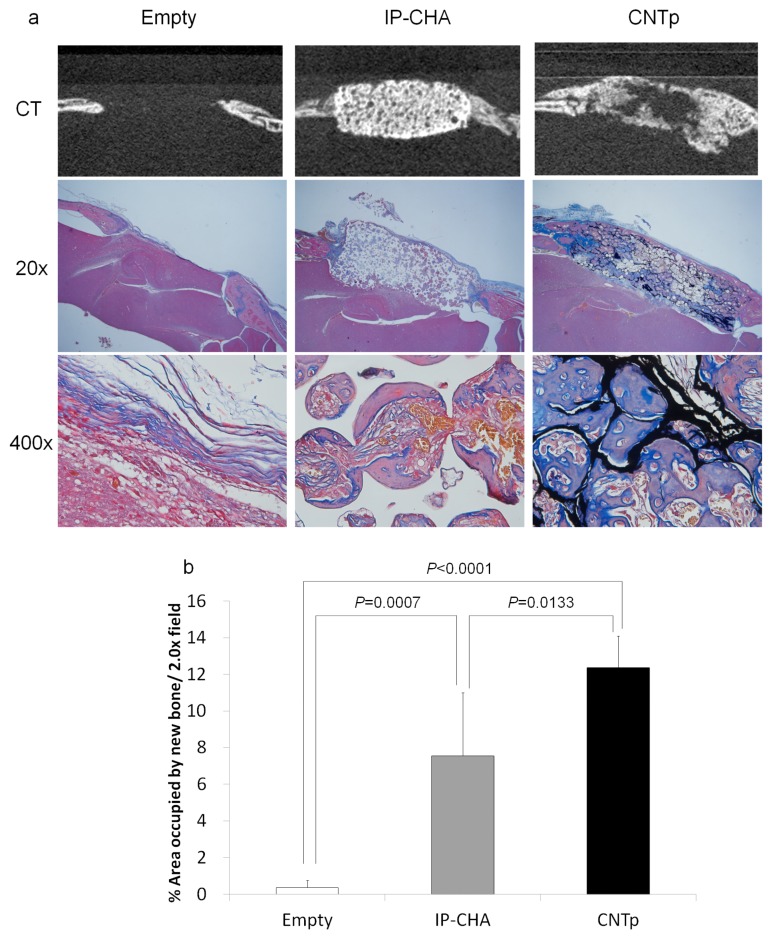
(**a**) Micro-CT and histological image of empty control, IP-CHA, and CNTp scaffolds combined with rhBMP-2, implanted in the calvarial defects of ddY mice for three weeks; (**b**) A bar diagram showing the newly formed bone area/20× field in the calvarial defect of ddY mice implanted with rhBMP-2 supplemented scaffolds. Mean values were compared using one-way ANOVA, followed by Tukey’s post-hoc test (*n* = 5).

Micro-CT analysis revealed that the new bone formation in the rhBMP-2 loaded CNTp group was comparable to that of the rhBMP-2 loaded IP-CHA group. The rhBMP-2 in saline (empty) group showed little new bone formation, and only membrane structures comprised of collagen fibers were observed. However, pores filled with woven and mature bones were observed in the rhBMP-2 loaded CNTp and IP-CHA groups (Figure 11a). CNTps showed a significantly larger area of newly formed bone in a 20× field (*p* < 0.0001 and *p* = 0.0133, respectively.) (Figure 11b).

## 3. Discussion

CNTs are considered to have many advantages, such as biocompatibility, strength, and ease of chemical modification [18]. On the other hand, the essential properties of bone regenerative scaffolds include biocompatibility, optimal pore size, and controlled release of growth factors [3]. We have previously reported excellent biocompatibility, bone forming abilities [19], and effect on osteoblasts [20] and osteoclasts [15] of powdery CNTs. Many researchers have evaluated CNT composite biomaterials for bone tissue engineering [17]. Significantly more in vivo bone formation was observed around the CNT-coated sponges than around the uncoated sponges [20]. PLGA/c-MWNT nanocomposites exhibited better adhesion, viability, and differentiation capacity [21]. On the CNT coated HA scaffold, unrestricted growth of human osteoblasts has been observed near CNT regions that are aided by CNT surfaces to promote cell growth and proliferation [22]. However, this is the first study that evaluated the capacity of a pure CNT scaffold with a well-controlled 3D structure for the use of bone regenerative medicine.

Crystals deposited on the surface of CNTps have a similar Ca/P ratio to HAs when being immersed in SBF. Fibrous CNTs are known to deposit HA when being immersed in SBF [12]. X-ray diffraction analysis (XRD) is often used to analyze crystals, including HA [23]. We attempted to perform XRD analysis, but it was impossible to detect the crystals that were deposited on the surface of CNTps alone. EDS (also called EDX: Energy Dispersive X-ray spectroscopy) is also commonly used to estimate the crystal component elements, and the Ca/P ratio of HA is known to be approximately 1.68 [24]. In this study, crystals observed on the surface of CNTps had a similar Ca/P ratio to HA and appeared to be HA or HA-like structures.

CNTs were known to be cytotoxic when internalized and accumulated in cells [25,26]. MWCNTs were also reported to be dissolved by macrophages, but the speed of dissolution is slow [27] and may remain in the body semipermanently. However, as we have previously reported, their toxicity in the body has been largely ignored [28,29]. In this study, because the CNTp is a well formed 3D architecture where CNTs are interconnected, there is not enough chance for individual CNTs to internalize in cells. This research demonstrated that the CNTp has low degradation and low cytotoxicity comparable to IP-CHA. Materials that have cytocompatible nano-structured surfaces are reported to promote better cell proliferation than conventional materials [30], but the optimal structure was different depending on the type of cells [31,32]. CNTp also has nano-scaled roughness on the surface of micro-pores, which benefits cell attachment and results in effective cell migration and growth.

In addition, CNTps showed good osteoconduction in the mouse calvarial bone defect model. HA crystals deposit around the type I collagen produced by osteoblasts in the bone regenerative process [33]. MWCNTs are considered to be osteocompatible by depositing HA that constructs the bone matrix in the location of bone regeneration.

CNTps showed lower Young’s modulus and energy to failure than IP-CHAs. Some cells show different migration and growth, depending on the stiffness of scaffold. It was reported that soft substrates promoted cell migration and rigid gels promoted osteogenesis [34]. Although CNTp was relatively softer than IP-CHAs, its rigidity was comparable to other 3D cells and was sufficient to support cell survival. Moreover, the CNTp showed strong adsorption of proteins and released them more gradually than the IP-CHA. In a BSA protein releasing study [35], the amount of BSA released from porous nano-hydroxyapatite/collagen/poly(l-lactic acid)/chitosan microspheres was approximately 80% of that of BMP, and the releasing kinetics of BSA were similar to that of BMP [11]. In the mouse calvarial defect model, rhBMP-2 loaded CNTps showed better bone regeneration than IP-CHAs. BMP can be immobilized and activate osteoblasts on the surfaces of various scaffolds [36]. CNT can also immobilize proteins on their surface [37]. In this research, CNTps adsorbed more and released less protein than IP-CHAs. The CNTps also showed the activation of MC3T3-E1 preosteoblast by loaded rhBMP-2 as well as the IP-CHAs. These facts suggest that BMP immediately immobilized on the surfaces of CNTps-activated cells and demonstrated better bone regeneration, and that bone regeneration using CNTps with rhBMP-2 is a compelling strategy for precise and enhanced bone repair. However, the true potential of CNTps for bone regeneration can only be realized through preclinical studies in large animal models. Moreover, the long-term safety and efficacy of regenerated bone in the body are still unclear. We are aiming to address these concerns in further research.

## 4. Materials and Methods

### 4.1. Sample Preparation

Multi-walled carbon nanotubes (MWCNTs, General Nano LLC, Cincinnati, OH, USA) have a diameter of approximately 10 nm, length of approximately 2 mm, and purity higher than 99%. Two different polymers were used in the foam fabrication processes: polymethyl-methacrylate (PMMA) microspheres (diameter: 100 ± 5 µm, sphericity: >99%, density: ~1.2 g/cc; Cospheric LLC, Santa Barbara, CA, USA) as a template for forming micro-pores, and polyacrylonitrile (PAN, Sigma-Aldrich, St. Louis, MO, USA) was used as a precursor to create graphene features among MWCNTs. The PMMA microspheres were used as a template for forming micro-pores, and PAN was used as a precursor to create graphene features among CNTs, which will increase the robustness of the CNT networks and also improve the attachment of the cells in the foam. The fabrication process involved adding a 1% PAN/dimethylformamide (DMF, 99.9% purity, EMD Millipore, Billerica, MA, USA) solution to 60 mg MWCNTs with a PAN/MWCNT weight ratio of 0.5. The mixture was well dispersed in 300 mL of isopropanol (IPA, VWR International, Radnor, PA, USA) by high power sonication. Then, 1200 mg of PMMA microspheres were added to the suspension, and the suspension was further sonicated in a bath sonicator for 10 min to achieve a uniform CNT/PAN/PMMA suspension. The CNT/PAN/PMMA composite was then obtained by a vacuum filtration method followed by drying in a vacuum oven to remove IPA and DMF. The porous structures were obtained through two heat treatments, first at 300 °C in air for 3 h and then at 1200 °C in nitrogen for 1 h. During the first heat treatment, the PMMA microspheres were depolymerized and expelled while the PAN precursor was stabilized. In the second high temperature treatment, the stabilized PAN precursor was carbonized to form graphene and nano-graphite features among MWCNTs [38,39]. The resulting blocks were termed CNT porous blocks (CNTps) and used in subsequent experiments. The CNTp has spherical micro-pores with 100 ± 5 µm in diameter and CNT networks with around 200 nm pores as the wall of the micro-pores. The surface roughness of the wall of micro-pores is in nanometer scale and there are micrometer scale channels between micro-pores, which benefit the cell attachment and migration. The density of the CNTp is 27 mg/cm^3^ and its porosity is 97%. The CNTp was cut into disks of 5 mm in diameter and 2 mm in height.

As a clinical control, we used IP-CHAs (NEOBONE, Covalent Materials Co., Tokyo, Japan) that are broadly applied in clinical medicine, featuring a pore size of 150 μm and porosity of 75%. In this study, we used test samples of the IP-CHAs that were cut into disks 5 mm in diameter and 2 mm in height.

### 4.2. Surface Observation by a Scanning Electron Microscopy

The pore size, distribution of pores, wall thickness, and interconnected pores of CNTps and IP-CHAs were observed by the Field Emission Scanning Electron Microscopy (FE-SEM, JSM-7600F; JEOL, Tokyo, Japan).

### 4.3. Mechanical Strength

Uniaxial compression tests were carried out on both the CNTp and IP-CHA (*n* = 3). Compressive strength was loaded against the bottom surface of the cylindrical scaffolds in a perpendicular direction by the Autograph AGS-H (Shimadzu Co, Ltd, Kyoto, Japan) at a compression speed of 1 mm/min.

### 4.4. Crystal Deposition Analysis

CNTps were immersed in 500 μL of r-SBF for 14 days, dried, and observed for deposition of crystals on their surfaces. FE-SEM was used to scan the surfaces and EDS was used for elemental analysis (*n* = 5). For comparison, IP-CHA without r-SBF treatment was scanned and analyzed.

### 4.5. Qualitative Protein Adsorption Profiles

CNTps and IP-CHAs were immersed in 500 μL of 500 μg/mL Bovine Serum Albumin (BSA, Wako Pure Chemical Industries, Osaka, Japan) solution for seven days. Subsequently, protein concentrations of these solutions were analyzed by a protein assay BCA kit (NacalaiTesque, Kyoto, Japan). Empty controls immersed no scaffolds in the solution.

### 4.6. In Vitro Protein Releasing Assay

10 μL of 50 mg/mL BSA solution was dropped (500 μg/sample) onto CNTps and IP-CHAs (*n* = 5), dried, and immersed in 500 µL of PBS (Nacalai Tesque, Kyoto, Japan). Supernatants were retrieved on day 1, 2, 3, 4, 5, 6, 7, and new PBS was added. Protein concentration of each supernatant was analyzed by a protein assay BCA kit (NacalaiTesque, Kyoto, Japan).

### 4.7. Degradation Assay

The degradation assay (based on the previous study [40]) was performed on CNTps and IP-CHAs over 21 days. Both scaffolds were weighed and then placed in 15 mL conical centrifuge tubes containing 3 mL 0.1 M Phosphate Buffered Saline (PBS) (pH 7.4) at 37 °C with constant shaking. The PBS was changed once a day. At days 1, 3, 5, 7, 14, and 21 scaffolds were removed from the tubes, dried in a desiccator overnight and weighed to determine weight loss over time.

### 4.8. Cell Culture

MC3T3-E1 preosteoblasts (RIKEN cell bank, Tsukuba, Japan) were cultured in alpha-modified minimum essential medium (alpha-MEM; Nacalai Tesque, Kyoto, Japan) with 10% heat-inactivated fetal bovine serum (Biowest, Nuaillé, France) and 1% Antibiotic-Antimycotic Mixed Stock Solution (NacalaiTesque, Kyoto, Japan) at 37 °C with 5% CO_2_ and 95% relative humidity. Media were replaced twice a week. Cells up to passage 20 were used.

### 4.9. Cell Morphology

The pattern of cell attachment on the surface of CNTp scaffolds were observed by fluorescence microscopy and SEM.

MC3T3-E1 preosteoblasts were trypsinized using a 0.25% trypsin/EDTA solution (Sigma, Saint Louis, MO, USA), seeded onto scaffolds in 96-well plates at a density of 1 × 10^4^ cells/scaffold, and suspended in 20 μL of media. They were incubated in a CO_2_ incubator for an hour at 37 °C and 5% CO_2_ saturated humidity until cells adhered to the scaffold. Each scaffold was then transferred to other 96-well plates and cultured by adding 200 μL of alpha-MEM. Media were replaced twice a week.

On day 5, scaffolds for fluorescence microscopic observation (*n* = 5) were fixed for an hour at 4% paraformaldehyde, treated an hour with 0.1% Triton-X, and stained with FITC-phalloidin (Sigma Aldrich, St. Louis, MO, USA) to show F-actin (red) + bisbenzimide H33342 fluorochrome trihydrochloride (Nacalai Tesque, Kyoto, Japan) and nuclei (blue) for an hour. After washing in PBS, they were observed with a fluorescence microscope (IX71, Olympus, Tokyo, Japan).

Scaffolds for SEM observation (*n* = 5) were fixed by 2.5% glutaraldehyde for 24 h, dried by 50–100% ethanol, sputter-coated by osmium, and observed by the FE-SEM (JEOL, Tokyo, Japan).

### 4.10. Cytotoxicity Determination

To evaluate cytotoxicity, MC3T3-E1 cells were seeded at a density of 5 × 10^4^ cells/scaffold onto CNTps and IP-CHAs placed in 48-well plates, and cultured for one and five days (*n* = 7 for each group). After staining by H33342, cells were observed by an IX71 inverted microscope at 400× magnification.

To compensate the limited field depth of 3D-culturing, multiple Z levels were integrated by the Zerene Stacker software (Zerene Systems, Richland, WA, USA). Consequently, cells in a single 400× field were counted.

Live/dead cell staining was done on the first and third day of incubation to visualize the cell viability. The cells were incubated with 0.1 µM Calcein-AM and 2 µM Ethidium homodimer-III (EthD-III) at 37 °C for 30 min (Live/Dead Cell Staining Kit II; PromoCell GmbH, Heidelberg, Germany). Stained cells were viewed using fluorescence microscopy (IX71, Olympus, Tokyo, Japan). Viable cells were stained with Calcein AM and fluoresced green, while dead cells were stained with EthD-III and fluoresced red.

### 4.11. Alkaline Phosphatase Activity Assay

MC3T3-E1 cells (1.0 × 10^4^ cells/scaffold) were seeded in a 48-well plate and cultured in alpha-MEM in the presence or absence of 100 ng/mL rhBMP-2 with a change of medium every three days. On differentiation day 7, the cells were washed with PBS twice and sonicated in the lysis buffer consisting of 0.1% Triton X-100. After centrifugation at 10,000× *g* for 5 min at 4 °C, the supernatant was transferred and the ALP activity was measured using a LabAssay ALP kit (Wako Pure Chemicals Industries, Osaka, Japan). The protein concentration of each sample was measured using a BCA Protein Assay kit (Nacalai Tesque, Kyoto, Japan). The relative activity of the sample is reported as the ratio of activity and the corresponding protein concentration (µmol/g). The significance was determined by Student’s *t* test.

### 4.12. Bone Regeneration of Mouse Critical-Sized Calvarial Defects

All experiments were carried out according to institutional guidelines for animal experimentation of Shinshu University School of Medicine. All protocols used in this study were reviewed and approved by the Division of Laboratory Animal Research (#260021). All surgery was performed under general and local anesthesia (intraperitoneal injection of sodium pentobarbital and subcutaneous injection of ridocaine), and all efforts were made to minimize suffering. All mice were euthanized using isoflurane inhalation at the end of the study.

Six-week-old male ddY mice (SLC, Shizuoka, Japan) were anesthetized with intraperitoneal injection of 30 mg/kg sodium pentobarbital (Kyoritsu Seiyaku, Tokyo, Japan) and 1 mL subcutaneous injection of 1% lidocaine (AstraZeneca, Osaka, Japan). Calvarial defects of 5 mm in diameter were made with a trephine bur, which were implanted with the CNTp and IP-CHA scaffolds. As a negative control, no scaffolds were implanted in the defects (Empty group) (*n* = 7 for each group).

After 12 weeks, the mice were euthanized by isoflurane (Abbott Japan, Tokyo, Japan) inhalation. After the heads were dissected, they were fixed in 10% formalin (Wako Pure Chemical Industries, Osaka, Japan), evaluated with µCT, and prepared for histological examination.

The µCT image of a mouse calvaria was collected at 80 kV tube voltage, 80 μA tube current, and scan time of 16 s. Raw data were reconstituted with *i*-View software (J. Morita, Kyoto, Japan) and analyzed.

Next, the tissues were decalcified in K-CX solution (Falma Co., Tokyo, Japan) for three days. After being embedded in paraffin, they were sliced into sections of 10 µm using a microtome. After being stained with Masson trichrome staining (Muto Pure Chemicals, Tokyo, Japan), sections were observed with an optical microscope (BX50; Olympus, Tokyo, Japan) (Masson trichrome-stained sections were used to define the collagen secreted in the process of bone formation, in which collagen fibers and woven bones are stained blue and mineralized bones are stained red [41]). Using a 2× objective lens, an image was captured to contain the entire bone defect. Then, the area of the new bone occupied in the 20× field was measured using ImageJ software (NIH, Bethesda, MD, USA) [42].

### 4.13. Bone Regeneration of Mouse Critical-Sized Calvarial Defects in Combination with Recombinant Human Bone Morphogenetic Protein-2 (rhBMP-2)

5 µg of 1 mg/mL rhBMP-2 (ATGen, Seongnam, South Korea) (equivalent to 5 µg of rhBMP-2) was added to the CNTp and IP-CHA scaffolds and dried for 24 h in order to prepare rhBMP-2-containing scaffolds. Six-week-old male ddY mice (SLC, Shizuoka, Japan) (*n* = 5 for each group) were anesthetized by the same method described in the previous section.

Calvarial defects of 5 mm in diameter were made with a trephine bur, which were implanted with rhBMP-2-containing scaffolds. As a control group, 100 µL of 0.05 mg/mL rhBMP-2 (corresponding to 5 µg of rhBMP-2) dissolved in normal saline (Otsuka Pharmaceutical Co., Ltd., Tokyo, Japan) was injected to the bone defect, after which wound closure was performed (Empty group).

After three weeks, the mice were euthanized and evaluated with μCT, examined histologically, and the area of the new bone occupied in the 20× field was measured by the same method described in the previous section.

### 4.14. Statistical Analysis

Data were expressed as mean and standard deviation. Each statistical analysis was performed using the Student’s *t* test, one-way ANOVA followed by Tukey’s post hoc test, and two-way ANOVA. Values of *p* < 0.05 were considered statistically significant.

## 5. Conclusions

In this study, we evaluated the capacity of the CNTp in the use of bone regenerative medicine. The CNTp is a highly porous material and has micro-pore size comparable to multi-pore 3D HA scaffolds that are clinically used in bone regenerative medicine. CNTp also had a good cytocompatibility, protein adsorption, and controlled protein release. In in vivo experiments, it showed excellent independent osteoconduction and also showed excellent new bone formation when loaded with rhBMP-2. From these results, we conclude that the CNTp is a promising scaffold that is not only a void filler for the treatment of bone fractures and bone defects but also a scaffold that allows cells to grow on, retain, or allow controlled release of growth factors to regenerate bones early and rigidly in the field of tissue engineering.
